# Gas–solid interfacial modification of oxygen activity in layered oxide cathodes for lithium-ion batteries

**DOI:** 10.1038/ncomms12108

**Published:** 2016-07-01

**Authors:** Bao Qiu, Minghao Zhang, Lijun Wu, Jun Wang, Yonggao Xia, Danna Qian, Haodong Liu, Sunny Hy, Yan Chen, Ke An, Yimei Zhu, Zhaoping Liu, Ying Shirley Meng

**Affiliations:** 1 Ningbo Institute of Materials Technology and Engineering (NIMTE), Chinese Academy of Sciences, Zhejiang 315201, China; 2 Department of Nano Engineering, University of California San Diego (UCSD), La Jolla, California 92093, USA; 3 Department of Condensed Matter Physics and Materials Science, Brookhaven National Laboratory, Upton, New York 11973, USA; 4 MEET Battery Research Center/Institute of Physical Chemistry, University of Müenster, Corrensstrasse 46, Müenster 48149, Germany; 5 Chemical and Engineering Materials Division, Oak Ridge National Laboratory, Oak Ridge, Tennessee, 37830, USA

## Abstract

Lattice oxygen can play an intriguing role in electrochemical processes, not only maintaining structural stability, but also influencing electron and ion transport properties in high-capacity oxide cathode materials for Li-ion batteries. Here, we report the design of a gas–solid interface reaction to achieve delicate control of oxygen activity through uniformly creating oxygen vacancies without affecting structural integrity of Li-rich layered oxides. Theoretical calculations and experimental characterizations demonstrate that oxygen vacancies provide a favourable ionic diffusion environment in the bulk and significantly suppress gas release from the surface. The target material is achievable in delivering a discharge capacity as high as 301 mAh g^−1^ with initial Coulombic efficiency of 93.2%. After 100 cycles, a reversible capacity of 300 mAh g^−1^ still remains without any obvious decay in voltage. This study sheds light on the comprehensive design and control of oxygen activity in transition-metal-oxide systems for next-generation Li-ion batteries.

## Abstract (web summary)

Oxygen activity can play a vital role in determining charge transport properties of materials. Here, the authors demonstrate a method to create oxygen vacancies on layered oxides via a gas-solid interface reaction, leading to materials with enhanced energy and power densities for Li-ion batteries.

## Graphical abstract

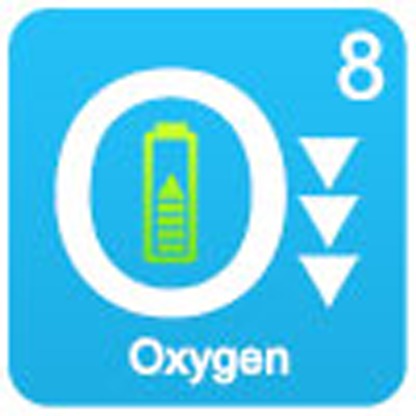

The functionality of many transition metal oxides can be significantly altered by oxygen vacancies on the surface. Oxygen vacancies can behave as charge carriers for solid-oxide fuel cells^1^, as well as important adsorption sites and as active sites for electro-photocatalysts^2^. In Li-ion cathode materials, these vacancies play a vital role in determining the material's electron and ion transport properties^3^,^4^,^5^. The influence of oxygen vacancies at the surface on electrochemical performance can be completely different depending on the type of Li-ion cathode material^6^,^7^,^8^. Li-rich layered oxides, either as a solid solution or as a nano-composite of layered Li_2_MnO_3_ and Li(TM)O_2_ (TM=Ni, Co, Mn), are drawing attention as next-generation cathode materials for high-energy-density Li-ion batteries in electric vehicles^9^,^10^,^11^. Over the past 20 years, the discharge capacity at room temperature of these cathode materials^9^,^12^,^13^,^14^ has been improved, from 200 mAh g^−1^, given in the first report^12^, to over 320 mAh g^−1^ (ref. 14) today as summarized by Hy *et al.*^15^, and even higher at elevated temperatures^16^. While research has continued to push the limit of the available capacity of the materials over the years, debates on the origins of the ultrahigh capacity beyond the redox of transition metal have intensified recently.

Numerous studies^10^,^17^,^18^,^19^,^20^,^21^,^22^,^23^ exemplify the contributions of bulk and surface oxygen on high charge–discharge capacity in Li-rich layered oxides, although this hypothesis has not been fully verified. Tarascon and co-workers^21^,^22^ concluded that the extra-high capacity is mainly attributable to reversible anionic redox processes (O^2−^/O_2_^2−^ or O^2−^/O_2_^n−^, where 3>*n*>1) within the bulk of Li-rich layered oxides. Delmas and co-workers^19^,^20^ proposed that surface oxygen is oxidized to O_2_ gas and irreversibly lost from the structure, leaving some oxygen vacancies on the surface or in the sub-surface layers. Our previous work^24^,^25^,^26^ illustrated that oxygen vacancies generated during the high-potential electrochemical process can facilitate the transition metal ion migration and surface structural transformation, finally leading to their potential degradation during extended cycles.

To reduce oxygen gas generation and utilize reversible oxygen redox activity during charging and discharging, oxygen vacancies have been proposed to form on the surface of the as-synthesized Li-rich layered oxides before electrochemical processing. The influence of the surface oxygen vacancies on the oxygen gas generation is discussed in detail in Supplementary Note 1. The creation of surface oxygen vacancies was previously attempted utilizing a reducing atmosphere^27^,^28^ and leaching with acid accompanied by heat treatment^29^,^30^. However, the bulk structure reported in previous work easily transforms from a pure layered phase to spinel- and/or rock-salt phases^28^,^29^,^30^,^31^,^32^,^33^, which diminishes the rate capability and cycling stability.

Motivated by the considerations above, we propose a strategy based on a gas–solid interface reaction (GSIR) between Li-rich layered oxides and carbon dioxide gas to create oxygen vacancies on the particles' surface. The schematic of GSIR process between Li-rich layered oxides and carbon dioxide is displayed in Fig. 1a, and the procedure is detailed in the ‘Methods' section. Oxygen vacancies in the surface regions up to 20-nm thick are created on the particles without affecting structural integrity. After the surface modification, the target material Li[Li_0.144_Ni_0.136_Co_0.136_Mn_0.544_]O_2_ exhibits discharge capacity as high as 300 mAh g^−1^ with no obvious voltage degradation after 100 cycles at a current density of 25 mA g^−1^. As confirmed by both theoretical calculations and experimental characterization, the improved electrochemical performance is ascribed to the full utilization of oxygen activity through the creation of oxygen vacancies on the surface of the as-synthesized Li-rich layered oxides.

## Results

### Characterizations of oxygen vacancies after GSIR process

Li-rich layered oxide Li[Li_0.144_Ni_0.136_Co_0.136_Mn_0.544_]O_2_ (denoted as LR-NCM) was prepared by a co-precipitation method (see the ‘Methods' section). After the GSIR process is completed, the general morphology remains the same as in the pristine LR-NCM (Supplementary Fig. 1). Both synchrotron X-ray diffraction (SXRD) refinements and transition metal K-edge X-ray absorption near-edge structure show no major difference for the pristine and GSIR LR-NCM samples (Supplementary Figs 2 and 3). These results preliminarily imply that the GSIR process does not heavily influence the average crystal structures and bulk electronic environments of the LR-NCM sample.

**Figure 1:**
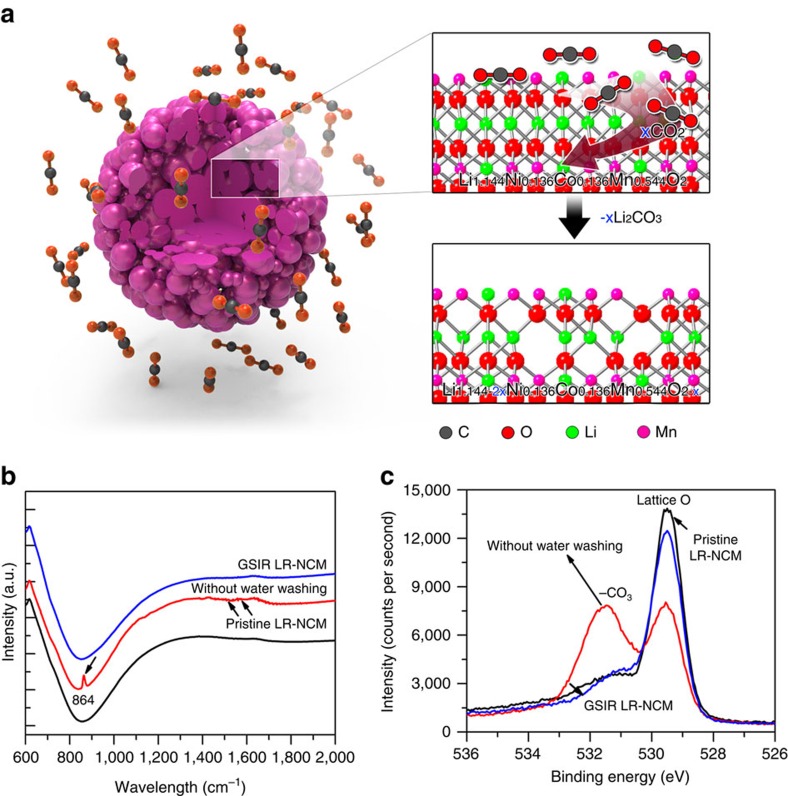
GSIR. (**a**) Schematic of GSIR between Li-rich layered oxides and carbon dioxide. (**b**) FTIR spectrum. The GSIR LR-NCM without water washing (red line), compared with the pristine LR-NCM, has three additional peaks, corresponding to the IR-active modes of –CO_3_. The sharp peak (864 cm^−1^) is the CO_3_ group bending mode, while the other two peaks (1,438 and 1,500 cm^−1^) are from the C–O asymmetric and symmetric stretching modes; (**c**) XPS spectra of the O 1s. It is clear that the GSIR LR-NCM has lower intensity than that of the pristine LR-NCM at lower-banding energy, which indicates some lattice O has been extracted by CO_2_.

To investigate the GSIR reaction mechanism of the formation of surface oxygen vacancies, we applied Fourier-transformed infrared spectroscopy (FTIR) and X-ray photoelectron spectroscopy (XPS) to three samples (Fig. 1b,c). The FTIR results (Fig. 1b) indicate that carbonate formed on their surfaces after the GSIR. The lithium concentration of the GSIR LR-NCM is slightly lower (6.4%) than that of the pristine LR-NCM, as was determined by inductive coupled plasma-atomic emission spectrometry. On the other hand, in the oxygen 1s XPS spectra (Fig. 1c), the intensity of the binding energy at 529.5 eV representing TM-O covalency for the GSIR LR-NCM after washing is much lower than that of the pristine LR-NCM. In contrast, the intensity of the binding energy at 531.5 eV representing the carbonate group (–CO_3_) for the GSIR LR-NCM without washing is much larger^34^,^35^. Both changes indicate some lattice oxygen was extracted by CO_2_, forming oxygen vacancies on the sub-surfaces.

**Figure 2:**
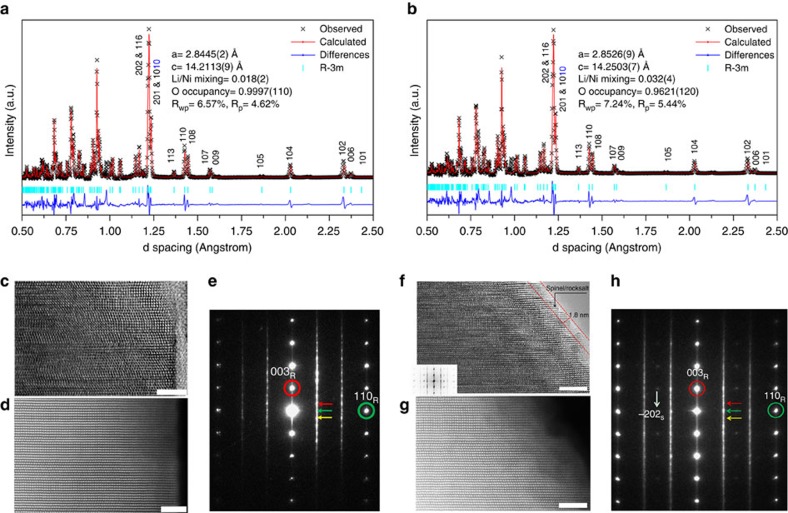
Structural characterizations of the pristine and GSIR LR-NCM. (**a**,**b**) TOF ND patterns for the pristine and GSIR LR-NCM. On the basis of these results, the oxygen vacancy for the GSIR LR-NCM is around three per cent higher than that of the pristine LR-NCM sample; (**c**) high-resolution transmission electron microscopy (HRTEM) image and, (**d**) high-angle annular dark-field—STEM (HAADF-STEM) image for the pristine LR-NCM; (**e**) electron-diffraction (ED) pattern for the pristine LR-NCM. The elongation of these spots are along [001]_R_* as well as [001]_M_* direction. The spots indicated with red, green and yellow arrows represent (110), (020) and (−110) of the monoclinic Li_2_MnO_3_ (donated as M) variants [1–10]_M_, [100]_M_ and [110]_M_, respectively. The spot marked by a red circle is shared by (003)_R_ of the rhombohedral (donated as R), as well as the structure (001)_M_ of all monoclinic variants. The spot marked by a green circle is shared by (110)_R_ of the rhombohedral, as well as the structure (33-1)_M_, (060)_M_ and (–331)_M_ of the monoclinic. (**f**) HRTEM image with fast Fourier transform (FFT) about the surface and (**g**). HAADF-STEM image for the GSIR LR-NCM; (**h**) ED for the GSIR LR-NCM. The weak spot indicated by the white arrow can only be indexed as the structure (−202)_S_ of the spinel (donated as S). Scale bars, 5 nm.

The amount of oxygen vacancies in the GSIR LR-NCM sample was determined by neutron diffraction (ND), a technique with high sensitivity for detecting light elements^36^, such as Li and O. Figure 2a,b demonstrates the as-collected time-of-flight (TOF) ND patterns with ‘Rietveld' refinement for both samples (patterns refined with a solid solution of ‘*R-3m*' symmetry; the normalized TOF ND patterns are shown in Supplementary Fig. 4). The lattice parameters of the pristine layer are a=2.8445(2) Å, and c=14.2113(9) Å. The oxygen occupancy of the pristine LR-NCM is 99.97±1.10%. In comparison, the lattice parameters of the GSIR LR-NCM are a=2.8526(8) Å and c=14.2503(7) Å, which are slightly larger than those of the pristine LR-NCM. The oxygen occupancy is reduced to 96.21±1.20% after surface modification. The amount of Ni^2+^ in the Li layer, and the occupancy of Li in the Li layer are also relatively larger than that of in the pristine LR-NCM. In addition, the data are also analysed using the two-phase model (the as-collected TOF ND patterns are shown in Supplementary Fig. 5), that is, a combination of LiTMO_2_ (*R-3m*) phase, and Li_2_MnO_3_ (*C/2m*) phase. The results show that no oxygen vacancies (V_O_) are observed in the pristine LR-NCM sample, while the GSIR LR-NCM sample exhibits 7.20±3.3% V_O_ on the 4i site and 3.8±3.0% V_O_ on the 8j site in Li_2_MnO_3_ phase and 2.96±1.86% V_O_ in the overall composition. Both models are consistent with the observed oxygen vacancies in the GSIR LR-NCM. The lower oxygen occupancy in the GSIR LR-NCM proves that the oxygen vacancies are successfully created after applying the GSIR process.

High-resolution transmission electron microscopy (TEM; Fig. 2c,f) was used to further illustrate the changes between the pristine and GSIR LR-NCM. A clean surface with Li and TM layers extending to the very edge of atomic planes is observed in the pristine LR-NCM (Fig. 2c). Meanwhile, a non-uniform surface (varying in thickness from 10 to 20 nm) is detected on the GSIR LR-NCM sample. In addition, high-angle annular-dark-field scanning-TEM images (Fig. 2d,g) for the pristine and GSIR LR-NCM are compared to demonstrate the lack of any obvious structural change. Although electron diffraction from some particles shows spinel/rock-salt-like phase in the GSIR LR-NCM compared with the pristine LR-NCM sample (Fig. 2e,h), it should be noted this phase transformation only occurs on the surface of GSIR LR-NCM within several atomic layers, as indicated in Fig. 2f, while the oxygen vacancy presence are 10–20-nm deep. These results are consistent with our SXRD and ND refinements, which verify that the GSIR process does not significantly alter the bulk structure.

**Figure 3:**
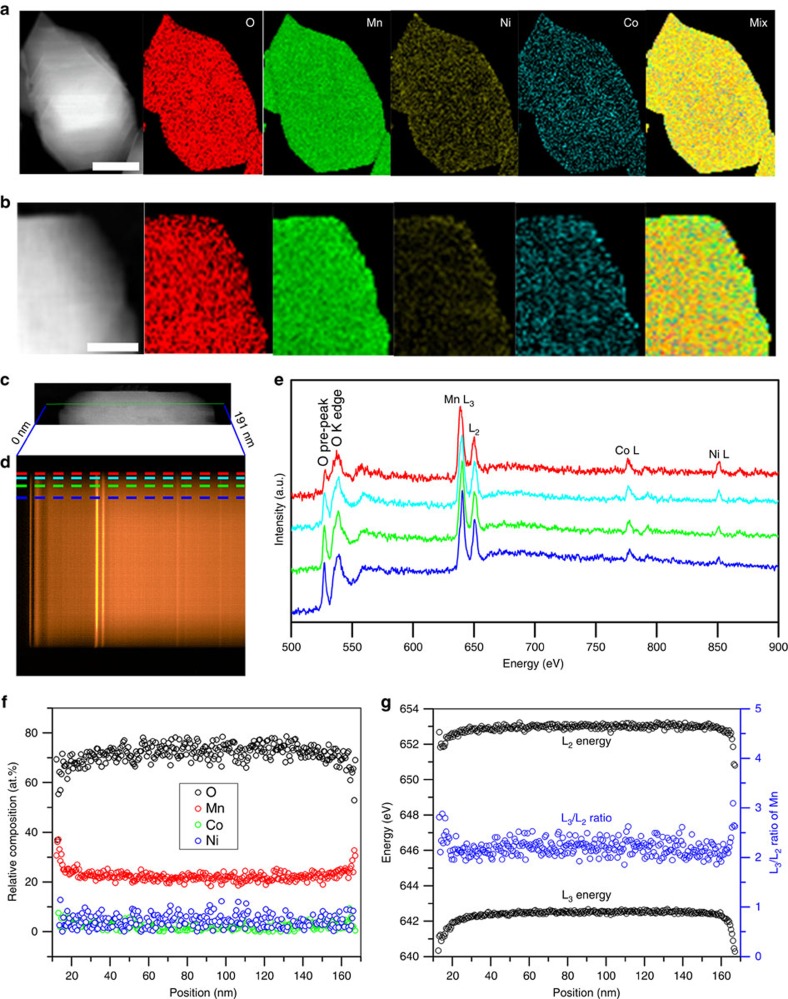
Relative surface composition changes for the pristine and GSIR LR-NCM. (**a**) STEM-EELS spectrum images for the pristine LR-NCM. Scale bar, 50 nm. (**b**) The GSIR LR-NCM. Scale bar, 25 nm. Oxygen reduction at mixed colour images is well-demonstrated on the edge in comparison with the pristine LR-NCM (**a**). (**c**) STEM image of a particle of the GSIR LR-NCM. (**d**) EELS spectrum image from the vertical green line (0–191 nm) in the left STEM image (**c**), showing O K, Mn L_2, 3_, Co L_2, 3_, Ni L_2, 3_ edges. The dispersion is 0.25 eV/channel. The scan step is 0.127 nm. (**e**) EELS spectrum profiles from the surface to the interior as marked by the horizontal dashed lines with the same colour as in **d**. The spectra are normalized with Mn L_3_ peak, and each spectrum was averaged vertically with 10 individual spectra to improve the signal noise ratio. Weak O pre-peak on the surface indicates the oxygen vacancies are formed. (**f**) Relative atomic composition of O (black), Mn (red), Co (blue) and Ni (green) as a function of position calculated based on the integrated EELS peak intensity in **e**. (**g**) Mn L_3_, and L_2_ energy (top and bottom of panel) from refinement, and L_3_/L_2_ intensity ratio.

Electron energy-loss spectroscopy (EELS) mapping was applied to compare relative surface composition changes for the pristine and GSIR LR-NCM sample (Fig. 3a,b). Oxygen reduction at mixed colour images is well-demonstrated on the edge of GSIR LR-NCM in comparison with the pristine LR-NCM. To study the electronic states of oxygen and transition metal from the particle, EELS was collected at different positions from bulk to surface of the GSIR LR-NCM (represented by colours in Fig. 3c,d). The spectra of the O-K-edge main peaks in Fig. 3e are normalized with the Mn L_3_ peak. Interestingly, it is observed that reduced O pre-peak intensities are from the surface in the GSIR LR-NCM sample. The weak pre-peak intensities can be even seen at 20 nm for the GSIR LR-NCM sample although the difference is small. The reduced ratio of the O-K-edge pre-peak intensities to the main peak can result from a change in the local environment of oxygen, especially from the oxygen vacancies formed on the surface^26^,^37^. The relative distribution of chemical composition is plotted in Fig. 3f. The content of O on average is 55% in the first 10 nm surface layer compared with the bulk value of 65%. The Mn L_3,2_ white lines provide information on Mn valence (oxidation); The Mn L_3,2_ white lines for the GSIR LR-NCM sample display a significant energy shift, and their L_3_/L_2_ intensity ratio remains low until 20 nm away from the surface of the GSIR LR-NCM sample (Fig. 3g), indicating a lower Mn valence on the particles' surface. When oxygen vacancies are created on the particles' surface, the valence of Mn is reduced simultaneously with the extraction of Li to compensate for the changes in charge. Therefore, as confirmed by both the ND and STEM/EELS results, oxygen vacancies have been successfully introduced on the 10–20 nm of the surface without a noticeable interruption in the bulk structure through the GSIR approach.

### Electrochemical properties of the pristine and GSIR LR-NCM

**Figure 4:**
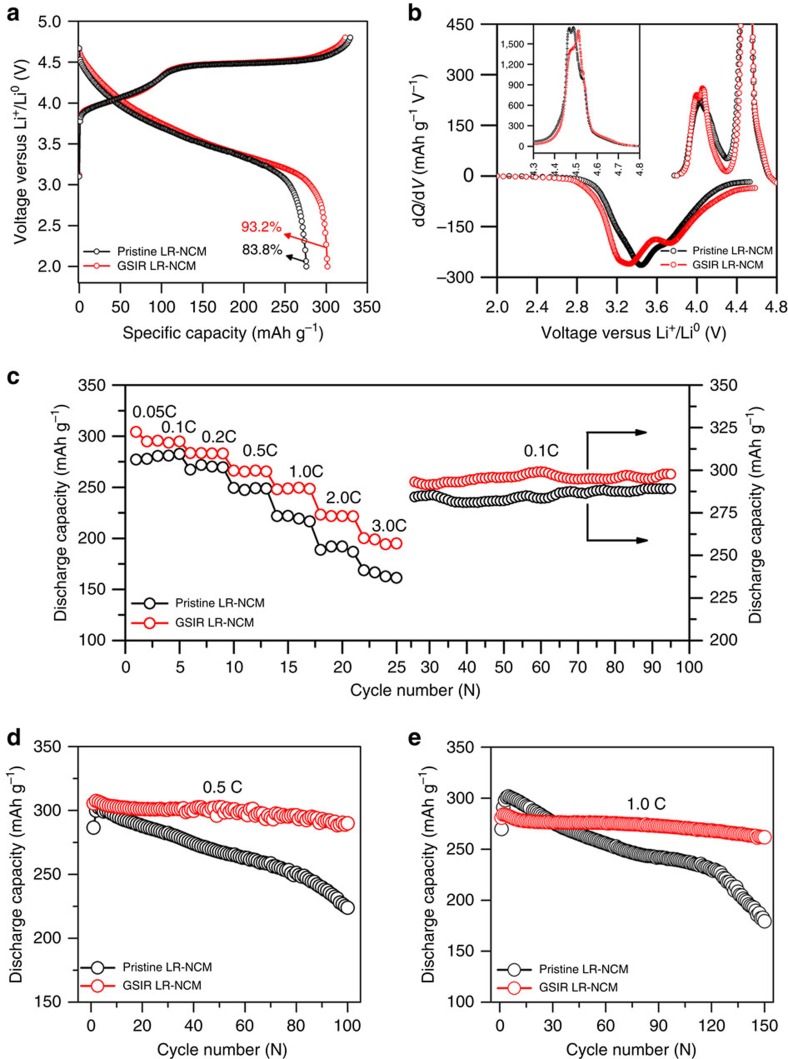
Charge–discharge characteristics of the pristine and GSIR LR-NCM. (**a**) First charge–discharge profiles of the pristine and GSIR LR-NCM obtained from a 2032-type coin cells at 0.05 C-rate, where 1.0 C-rate corresponds to the current density of 250 mA g^−1^. (**b**) Differential capacity (d*Q/*d*V*) plots of the initial cycle curves of the pristine and GSIR LR-NCM. The inset shows the enlarged d*Q/*d*V* curves about 4.5 V. (**c**) Discharge-rate capacity after charging galvanostatically at 0.1 C-rate before each discharge. The capacity retention when performing charge–discharge cycles at constant 0.1 C-rate for 70 cycles after all rates tested. (**d**,**e**) Cycling performance of the pristine and GSIR LR-NCM at 55 °C by applying a constant current density of 0.5 C-rate and 1.0 C-rate (250 mA g^−1^), respectively. The loading density of the active material on the electrode was around 5.5 mg cm^−2^. It is noticeable that the electrochemical data are derived from the average value of at least five coin cells, and the errors on the specific capacity are about 3 mAh g^−1^.

CR2032 coin cells with metallic Li as the counter electrode were assembled to investigate the electrochemical performance of the pristine and GSIR LR-NCM samples. Similar to other Li-rich layered oxides, both of them (Fig. 4a) exhibit a long plateau region at ∼ 4.5 V versus Li^+^/Li^0^, which usually is ascribed to the electrochemical activation of the Li_2_MnO_3_ component during the initial charge process^38^,^39^,^40^, although an electrolyte/electrode side reaction is expected in this region. Interestingly, the value of the initial discharge capacity for the GSIR LR-NCM can reach as high as 301 mAh g^−1^ compared with that of 276 mAh g^−1^ for the pristine LR-NCM. The initial coulombic efficiency also increases from 83.8 to 93.2%. The corresponding differential capacity versus voltage (d*Q/*d*V*) curves are plotted in Fig. 4b. A lower shift in the oxidation peak after the GSIR process is due to the decrease in electrochemical impedance (Supplementary Fig. 6 and Supplementary Table 1). In addition, the decline of the oxidation peak around 4.5 V versus Li^+^/Li^0^ observed in the enlarged d*Q/*d*V* curves (inset in Fig. 4b) indicates that the GSIR process has pre-activated the Li_2_MnO_3_ component responsible for the 4.5 V plateau. The rate capability and cycling stability further highlight the advantages of our GSIR LR-NCM sample (Fig. 4c). At all tested rates, the GSIR LR-NCM exhibits a higher capacity than that of the pristine LR-NCM. The unique characteristic at different rates for the GSIR LR-NCM is that the additional discharge capacity (Supplementary Fig. 7a–f) results only from the lower-potential region (<3.5 V versus Li^+^/Li^0^). It is remarkable that the GSIR LR-NCM delivers a higher discharge capacity of 298 mAh g^−1^ when it returns to the 0.1 C-rate, compared with that of 288 mAh g^−1^ for the pristine LR-NCM. More importantly, the charge–discharge plots at subsequent cycles (Supplementary Fig. 7g,h) for the GSIR LR-NCM demonstrate a slight degradation in potential after 100 cycles, even for a discharge capacity as high as 300 mAh g^−1^. To track the origin of the additional capacity after the GSIR, the energy density and discharge capacity below and above 3.5 V versus Li^+^/Li^0^ are plotted as a function of the cycle number, as depicted in Supplementary Fig. 8i,j. It proves that the additional capacity in discharge capacity also comes from the lower-potential region (<3.5 V versus Li^+^/Li^0^).

To evaluate the stability of the GSIR LR-NCM, a more challenging measurement was selected (Fig. 4d,e). Cells based on the GSIR LR-NCM show a higher initial capacity of 306 mAh g^−1^ (0.5 C-rate) and 280.9 mAh g^−1^ (1.0 C-rate), compared with that of 287 mAh g^−1^ (0.5 C-rate) and 269 mAh g^−1^ (1.0 C-rate) for the pristine LR-NCM at the elevated temperature of 55 °C. The initial charge–discharge curves (Supplementary Fig. 8a,b) are similar to the results at room temperature (Fig. 4a). In addition, the cells based on the pristine LR-NCM display only 223 mAh g^−1^ (at 0.5 C-rate) and 179 mAh g^−1^ (at 1.0 C-rate) after 100 cycles and 150 cycles, respectively, whereas the GSIR LR-NCM shows an excellent capacity of about 290 mAh g^−1^ (at 0.5 C-rate) and 262 mAh g^−1^ (at 1.0 C-rate) during the same cycling period. Moreover, the charge–discharge plots of subsequent cycles (Supplementary Fig. 8d,f) for the GSIR LR-NCM exhibit much slower potential degradation profiles at different rates than those of the pristine LR-NCM (Supplementary Fig. 8c,e). The difference clearly signifies that surface oxygen vacancies introduction in Li-rich layered oxides without severe structural destruction has a considerable effect on improving electrochemical performance.

## Discussion

The influence of surface oxygen vacancies introduced by the GSIR process on the electrochemical performance can be discussed in two aspects. (1) The generation of highly reactive oxygen radicals on the particles' surface during the electrochemical process, which determines the electrochemical resistance at the electrode/electrolyte interface; (2) the participation of the oxygen reaction in the form of O^2−^/O^−^ or O_2^2−^_ in the bulk of the material, which are the dominant factors in compensating for extra electrons on the superior capacity of Li-rich layered oxides.

**Figure 5:**
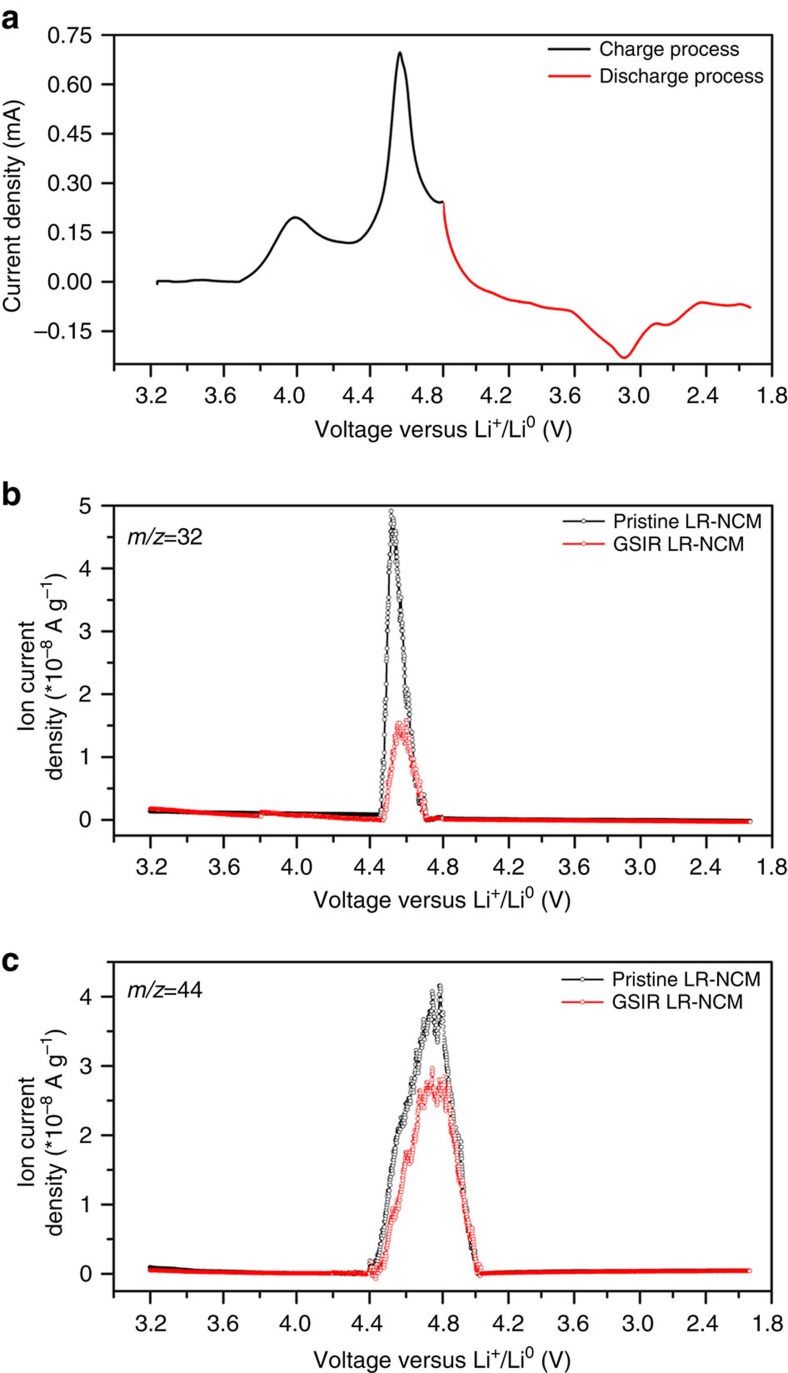
*Operando* DEMS. (**a**) Initial cyclic voltammetry (CV) curve of the pristine and GSIR LR-NCM electrode. (**b**) Oxygen gas profile of the initial charge–discharge process for the pristine and GSIR LR-NCM electrode. (**c**) Carbon dioxide gas profile of the initial charge–discharge process as the above electrodes. The loading density mass for these two electrodes are the same about 7.76 mg. The scan rate was ∼0.1 mV s^−1^, and the potential range was between 2.0 and 4.8 V versus Li^+^/Li^0^ at an elevated temperature of 55 °C.

First, *operando* differential electrochemical mass spectrometry (DEMS) experiments were performed to evaluate the gas evolution during the initial charge–discharge process (Fig. 5a), which is largely related to the activity of oxygen on the particles' surface^18^,^41^,^42^. Irrespective of the cycling profile, as shown in Fig. 5b, O_2_ is detected near 4.5–4.6 V versus Li^+^/Li^0^ for both the pristine and GSIR LR-NCM; however, the O_2_ gas released from the GSIR LR-NCM electrode is much less than that of the pristine electrode. It is suggested that the formation of oxygen vacancies through GSIR could greatly suppress the evolution of O_2_ gas. According to the equation 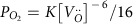 in the Supplementary note 1, less O_2_ gas will be released due to the reduced partial pressure of oxygen on the surface of the GSIR LR-NCM electrode after oxygen vacancies are created. At the same time, the released CO_2_ gas for the GSIR LR-NCM (Fig. 5c), which is usually considered as the source of decomposition of the electrolyte at high potentials^42^, is smaller than that of the pristine LR-NCM. Therefore, the oxygen vacancies on the surface can hinder the highly reactive oxygen radicals from generating during the electrochemical process.

**Figure 6:**
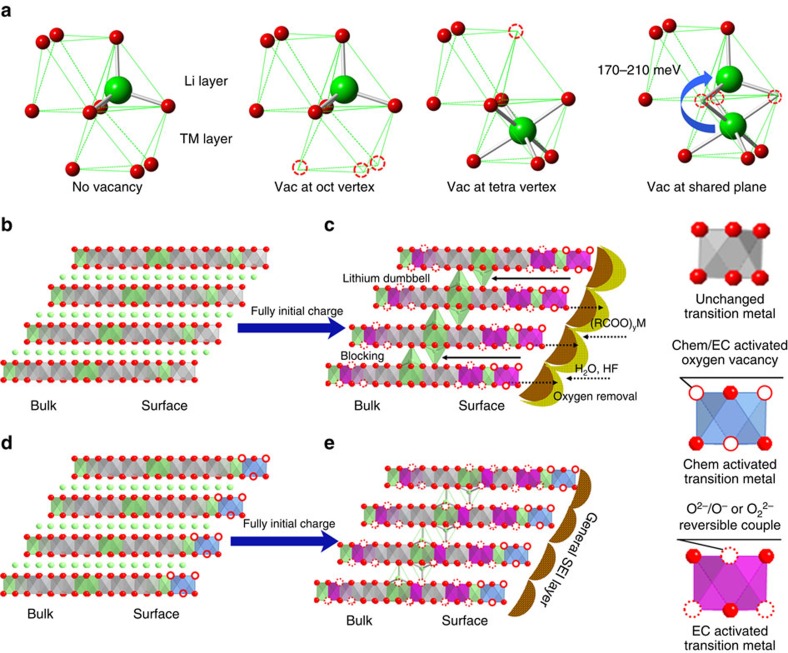
Reaction mechanisms during charging and discharging. (**a**) Calculated site stability and activation barrier under conditions of no vacancy, vacancy at octahedron (Oct) vertex, vacancy (Vac) at tetrahedron (Tetra) vertex and vacancy (Vac) at shared plane. (**b**,**c**). The pristine LR-NCM before charging and after full initial charge. (**d**,**e**) The GSIR LR-NCM before charging and after full initial charge. Green, Li; red, O. The fundamental contribution of the novel surface modification is the uniform creation of lithium and oxygen vacancies on the surface of particles before electrochemical cycling. Consequently, more Mn^4+^/Mn^3+^ redox couples in Li_2_MnO_3_ component are pre-activated; less oxygen gas is released from the material and less oxygen vacancies are created in the bulk during the charging process; limited electrode/electrolyte corrosion and bulk material structure transformation are formed in the battery system.

Second, the GSIR LR-NCM sample exhibits almost the same capacity in the plateau region as in the pristine sample with much less oxygen redox participation on the surface. To compensate for ionic changes during the charge process, more oxygen reactions in the form of O^2−^/O^−^ or O^2−^ (instead of the O^2−^/O_2_ redox couple) are triggered in the bulk of the GSIR LR-NCM. The influence of the bulk oxygen activity in the diffusivity of lithium ions was investigated by first-principles calculations. For simplicity, the supercell was defined as Li_28_Ni_6_Mn_14_O_48_, which was adopted as a computational model in the previous work^24^. A specific Li_20/28_ concentration was chosen to simulate the last stage of discharge (below ∼3.2 V), in which the GSIR LR-NCM demonstrates the biggest difference in electrochemical performance compared with the pristine LR-NCM. This stage is also generally acknowledged as a rate-determining step of the whole discharge process in Li-rich layered oxides^43^. In this stage, the Li-ion migrates from the octahedral site of the Li layer into the octahedral site of the adjacent transition metal layer through an empty tetrahedral site in the former (as shown in Fig. 6a). When no oxygen vacancy is introduced, the Li tetrahedron site, which is face-sharing with the TM layer Li, is most stable. There is a high chance of trapping the Li-ion in the tetrahedral site (so-called ‘Li–Li dumbbell') without diffusing into the adjacent octahedral site in the transition metal layer^24^. These dumbbells finally block the pathways of lithium diffusion, thereby decreasing ionic conductivity. In contrast, when oxygen vacancies are introduced the under-coordinated site is no longer stable, and the migration barrier is either zero or only around 170–210 meV (ref. 44). The trapped Li-ion has a higher chance of escaping from the tetrahedral sites and continuing its diffusion process.

Overall, Fig. 6b,e summarizes the proposed reaction mechanism for the pristine and GSIR LR-NCM during charging and discharging. Before charging, uniform oxygen and lithium vacancies are created on the particle surface of the GSIR LR-NCM, accompanied by the pre-activation of certain amount of the Li_2_MnO_3_ component. The sloped region (<4.4 V) of the charging curve is attributed to the extraction of Li^+^ ions from the lithium layer. No difference is expected between the pristine and GSIR LR-NCM material during this process. During the charging plateau region, more lithium ions can be extracted from the Li_2_MnO_3_ component, together with the oxidation of lattice oxygen^19^,^20^,^22^,^23^. The pre-activated surface layer of the GSIR sample with oxygen vacancies reduces the partial pressure of oxygen on the surface, and then prevents gaseous oxygen from evolving during plateau charging. A thinner SEI layer is thus expected to form on the particles' surface of the GSIR LR-NCM due to less side reactions between released oxygen and the electrolyte species, thereby decreasing the electrochemical resistance at the electrode/electrolyte interface, as supported by our electrochemical impedance-spectroscopy (EIS) results (Supplementary Fig. 6 and Supplementary Table 1). At the same time, the more reversible oxygen redox-reaction is triggered in the bulk of the GSIR LR-NCM during the high-potential-charging process. These oxygen vacancies can activate the Li trapped in the tetrahedral sites to provide a favourable environment for Li-ion diffusion during the rate-determining step (the last stage of discharging). Higher levels of Li-ion diffusivity in the bulk, together with smaller resistance to charge-transfer and larger numbers of activated Mn^4+^/Mn^3+^ couples lead to higher capacity and better rate capability of the GSIR LR-NCM sample. Furthermore, minimal structure changes enable the superior cycling stability after GSIR modification.

In summary, a facile but effective approach to surface modification based on GSIR has been applied to create oxygen vacancies homogeneously without causing structural destruction. Oxygen vacancies in the surface regions up to 20-nm thick are found to facilitate Li diffusion by activating the Li in the tetrahedral sites and suppress the release of gas from the surface, which finally lead to higher discharge capacity and a better rate capability in the Li-rich layered oxides. To the best of our knowledge, this study also clarifies for the first time the critical influence of the activity of surface oxygen in enhancing the electrochemical performance. We believe this facile, scalable and inexpensive approach opens a door for comprehensive designing and controlling oxygen activity in transition-metal-oxide systems to achieve excellent energy and power density in their commercial applications.

## Methods

### Synthesis of Li[LO_0.144_Ni_0.136_Co_0.136_Mn_0.544_]O_2_

The synthesis procedures of Li[LO_0.144_Ni_0.136_Co_0.136_Mn_0.544_]O_2_ were detailed as follows^45^: we pumped an aqueous solution containing NiSO_4_·6H_2_O, CoSO_4_·7H_2_O and MnSO_4_·4H_2_O with a concentration of 2.0 mol l^−1^ into a continuously stirred tank reactor (CSTR, capacity of 50 l); at the same time, a 2.0 mol l^−1^ Na_2_CO_3_ solution and a 0.2 mol l^−1^ NH_4_OH solution were added separately into the reactor. The co-precipitation temperature was held at 60 °C, and the pH value was fixed to 7.8. The resulting (Ni_1/6_Co_1/6_Mn_4/6_)CO_3_ powders were washed several times with distilled water to remove residual Na^+^, and dried in a vacuum oven at 80 °C for over 20 h. The resulting precipitates were mixed with Li_2_CO_3_ and the exact molar ratio between them was 0.7. The mixed powders were first pre-treated at 500 °C for 5 h in the air and then calcinated at 850 °C for 15 h in the air. Then, they were cooled to room temperature in the furnace. The as-obtained sample was labelled as pristine LR-NCM.

### GSIR process

The GSIR process of the pristine LR-NCM sample with CO_2_ was carried out as follows: a set amount of LR-NCM cathode materials and a given amount of NH_4_HCO_3_, which is as the source of CO_2_ after decomposition at certain temperatures, were placed in an enclosed reactor in an argon-filled glove box (O_2_<0.1 p.p.m.) with a total volume of 100 ml, and heated at 200 °C for 10 h. The optimal molar ratio between carbon dioxide and Li-rich layered oxides was about 1:5. It is important that the pristine LR-NCM cathode materials and the NH_4_HCO_3_ must be separated at the beginning for ensuring a homogeneous GSIR. To remove the reaction products on the surface of the LR-NCM, the sample obtained was washed with water several times and dried at 120 °C for 12 h. The as-obtained product was termed the GSIR LR-NCM. We would like to stress that the same procedures have been adopted in both NIMTE and UCSD labs, and all the electrochemical data have been completely reproduced.

### Materials characterization

A field-emission scanning electron microscopy image was acquired on a Hitachi S-4800. A FTIR analysis was performed on a pellet made of active materials and KBr powder, using an FTIR spectrometer (Nicolet, 6700 series, USA). XPS measurements were collected using an AXIS Ultra DLD spectrometer with Al Kα (1,253.6 eV) radiation, to investigate the changes in the compositions on the cathode surface. The compositions of the pristine, and GSIR LR-NCM sample were confirmed quantitatively by inductive coupled plasma-atomic emission spectrometry with an emission spectrometer (Optima 2100 DV, Perkin-Elmer). The tap density of the pristine LR-NCM sample was measured and calculated by tap density instrument (FSZ-4, Ruike).

### TOF powder ND

The TOF powder ND data were collected on the VULCAN beamline at the Spallation Neutron Sources (SNS) in the Oak Ridge National Laboratory^46^. Around 0.6 g of powder was packed into a vanadium sample can. An incident beam (5 mm × 12 mm) of 0.7- to 3.5-Å bandwidth allowing a 0.5–2.5 Å d-spacing in the diffracted patterns in the±90° 2θ detector banks was selected, using the double-disk choppers at a speed of 30 Hz. A high-resolution mode was used with Δd/d ∼0.25%. The SNS was at a nominal 1,100 kW. Powder-neutron-diffraction data were collected at a high-resolution mode for 3 h and reduced by the VDRIVE software. Full-pattern Rietveld refinement was performed using the GSAS programs with the EXPGUI interface^47^.

### TEM characterization

TEM images and EELS spectra were carried out using the JEM-ARM200CF scanning/transmission electron microscope at Brookhaven National Laboratory. The instrument is equipped with a cold field-emission source, two aberration-correctors and a high-resolution dual electron energy-loss spectrometer (Quantum GIF). The optimal energy-resolution was ∼0.35 eV, as judged by the full-width at half-maximum of the zero-loss peak. Spectra were recorded either using a convergent-beam in STEM mode, or parallel-beam in TEM mode, for diffraction, imaging and spectroscopy. The refinement Mn L_3_, and L_2_ energy, and L_3_/L_2_ intensity ratio were based on Pearson's method^48^.

### X-ray diffraction and X-ray absorption spectroscopy

SXRD data were collected using beamline BL14B1 at the Shanghai Synchrotron Radiation Facility (SSRF). These data were recorded in the 2θ range between 10^o^ and 85^o^, using a fixed wavelength of 1.2398 Å. The structural composite study was carried out by Rietveld refinement analysis, using the GSAS program. For X-ray absorption fine-structure spectroscopy studies, we performed measurements at beamline BL14W1 of the SSRF. The incident beam was mono-chromatized using a Si (111) fixed-exit, double-crystal monochromator. Foils of nickel, manganese and cobalt were used for energy calibration with zero energy (*E*_*0*_), defined according to Kraft *et al.*^49^. Spectra were acquired in transmission mode, utilizing gas-ionization chambers as detectors. Data on X-ray absorption near-edge structure was extracted with established methods using the ATHENA software package.

### Electrochemical measurements

The negative electrodes were prepared by casting the slurry with a mixture of 80 wt.% the pristine or GSIR LR-NCM, 10 wt.% acetylene black and 10 wt.% polyvinylidene fluoride binder on an aluminium foil. Electrode discs of 13-mm diameter were punched from the negative electrode, pressed at 8 Mpa per disc and dried at 80 °C for 12 h in vacuum to remove any residual NMP and traces of water. The prepared electrodes then were assembled in an Ar-filled glove box (H_2_O<0.1 p.p.m. and O_2_<0.1 p.p.m.). Metallic Li was used as the counter electrode, and Celgard 2502 as a separator. The electrolyte solution was 1 M LiPF_6_ in a 3:7 (volume ratio) mixture of ethylene carbonate–dimethyl carbonate (Zhangjiagang Guotai-Huarong New Chemical Materials Co., Ltd.). The cells were galvanostatically charged and discharged on a LAND-CT2001A battery-test system. Unless otherwise specified, the cells typically cycled between 2.0 and 4.8 V versus Li^+^/Li^0^, and the temperature for testing was about 28 °C. EIS measurements were performed on an Autolab83710 impedance analyser with a.c. voltage amplitude of 5 mV, and frequency range of 0.01–100,000 Hz. The metallic Li served as both the counter and reference electrodes during the EIS measurements.

### *Operando* DEMS

*Operando* DEMS analysis was carried out to detect the gases generated during the initial charge–discharge process. The DEMS cell was assembled in an Ar-filled glove box. In all, 1 M LiPF_6_ in 1:1 ethylene carbonate:dimethyl carbonate was used as the electrolyte, and a glass-fiber filter (Whatman GF/D) was used as a separator. Electrodes were prepared by casting a slurry with a composition of 80 wt. % active materials, 10 wt. % Super C65 (Timcal) and 10 wt.% PVDF (Kynar FLEX 761A, Arkema Group) on to an aluminium current-collector foil. Electrode tapes then were punched into 22-mm diameter discs, and dried at 120 °C in an under vacuum oven for 24 h. The electrodes were assembled into Swagelok-type cells, using metallic Li as both the counter and the reference. Thereafter, the cell was connected to the mass spectrometer. The cell was purged continuously with gaseous Ar, which flowed from the cell into the mass spectrometer carrying the evolved gases for MS analysis. *Operando* DEMS cells were conducted on a VSP electrochemical workstation (Biologic, France). The mass signals were recorded as a function of time and the cell voltage. The time resolution of ion-current intensity is optimized by selectively scanning the *m/z* 32 and 44 signals. In line with previous studies^41^,^42^, only O_2_ and CO_2_ were evolved during the initial cycling of Li-rich layered-oxide electrodes.

### Computation methodology

First-principles calculations were performed in the spin-polarized GGA+U approximations to the density functional theory. The core electron states were represented by the projector-augmented-wave method^50^ as implemented in the Vienna *ab initio* simulation package^51^,^52^,^53^. We used the Perdew–Burke–Ernzerhof^54^ exchange correlation, and a plane-wave representation for the wave function with a cutoff energy of 450 eV. The Brillouin zone was sampled with a dense k-points mesh by Gamma packing. Our supercell, composed of 24 formula units of Li[Ni_1/4_Li_1/6_Mn_7/12_]O_2_, used in our previous work was used again to obtain the site's stability and activation barrier. The Hubbard U correction was introduced to describe the effect of localized d electrons of the transition-metal ions. The applied effective U value given to Mn ions is 5 eV, and that to Ni ions is 5.96 eV (ref. 55). In this calculation, a concentration of Li20/28 was applied, in which the GSIR LR-NCM exhibits a difference in electrochemical performance compared with pristine LR-NCM. The nudged elastic band method was used to find the minimum-energy path and the energy barrier for Li diffusion inside the materials.

### Data availability

The data that support the findings of this study are available from the corresponding authors on request.

## Additional information

**How to cite this article:** Qiu, B. *et al.* Gas–solid interfacial modification of oxygen activity in layered oxide cathodes for lithium-ion batteries. *Nat. Commun.* 7:12108 doi: 10.1038/ncomms12108 (2016).

## Supplementary Material

Supplementary Information Supplementary Figures 1-8, Supplementary Table 1, Supplementary Note 1 and Supplementary References
