# Supplementary material for: Gas–solid interfacial modification of oxygen activity in layered oxide cathodes for lithium-ion batteries
Source: Nat Commun. 2016 Jul 1;7:12108. doi: 10.1038/ncomms12108 (PMC4932185; doi:10.1038/ncomms12108)
Supplement: Supplementary Information — Supplementary Figures 1-8, Supplementary Table 1, Supplementary Note 1 and Supplementary References [file ncomms12108-s1.pdf]

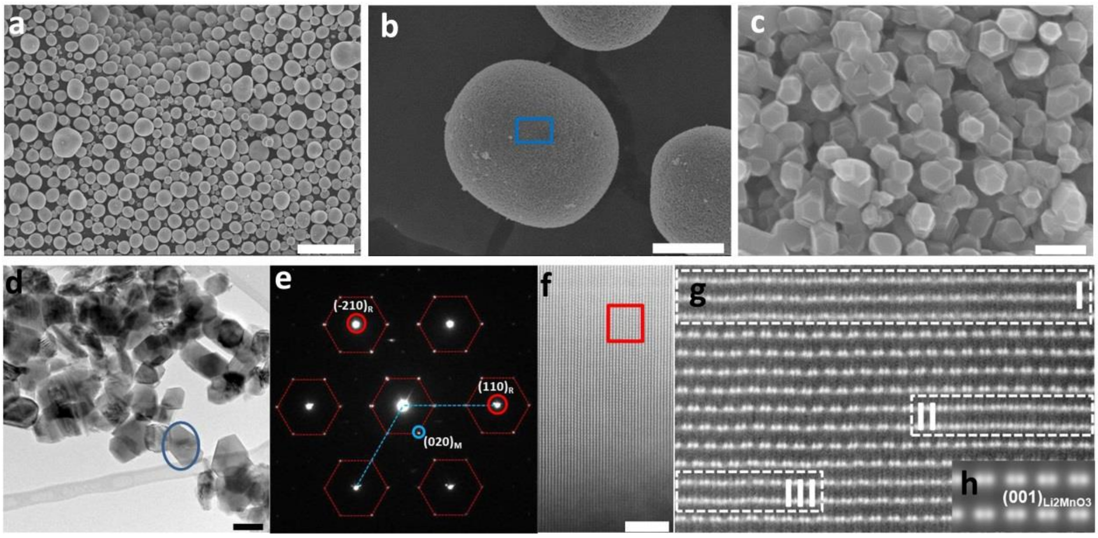

Supplementary Figure 1| Morphology and structure of the pristine LR-NCM. a-c. Scanning electron microscopy (SEM) images at different magnifications for the pristine LR-NCM. The scale bars are 50 µm, 5µm and 200nm, respectively. The as-prepared cathode material exhibits typical spherical morphology with an average particle size of 13 µm, where the secondary particles are aggregated by the primary nanoparticles with the size between 100 nm and 200 nm. From a practical application perspective, the cathode material with micrometer-sized spherical morphology has high tap density and volumetric energy density. The tap density was measured about 2.2 g cm^{-3}. **d.** Transmission electron microscopy (TEM) bright-field image showing that the particle size is about 100 nm. The scale bar is 100 nm; **e.** electron diffraction pattern (EDP) from a particle circled in (**d**), displaying (001)* zone of the R phase and the M phase; **f**-**g**. High-angle annular dark-field (HAADF) – Scanning TEM (HAADF-STEM) images for the pristine LR-NCM. The scale bar is 5 nm; because the image intensity of each atomic column is proportional to the average atomic number ($\sim Z^{1.7}$)[1], the bright spots are resulted from the heavy atomic columns (Ni, Co, and Mn) in the pristine LR-NCM, while light atoms (Li and O) are nearly invisible in the image showing dark dot contrasts. It can be observed that there are some areas with continuous bright dots contrasts (area I, II, and III) and some other areas with discontinuous bright dots contrasts; **h.** HAADF image calculated based on the monoclinic Li_2MnO_3 structure, which agrees with the experimental image. Both indicate that this Li-rich layered material can be considered to be a mixture of

nanosized rhombohedral $LiMO_2$ and monoclinic Li_2MnO_3 components in a single crystal particle.

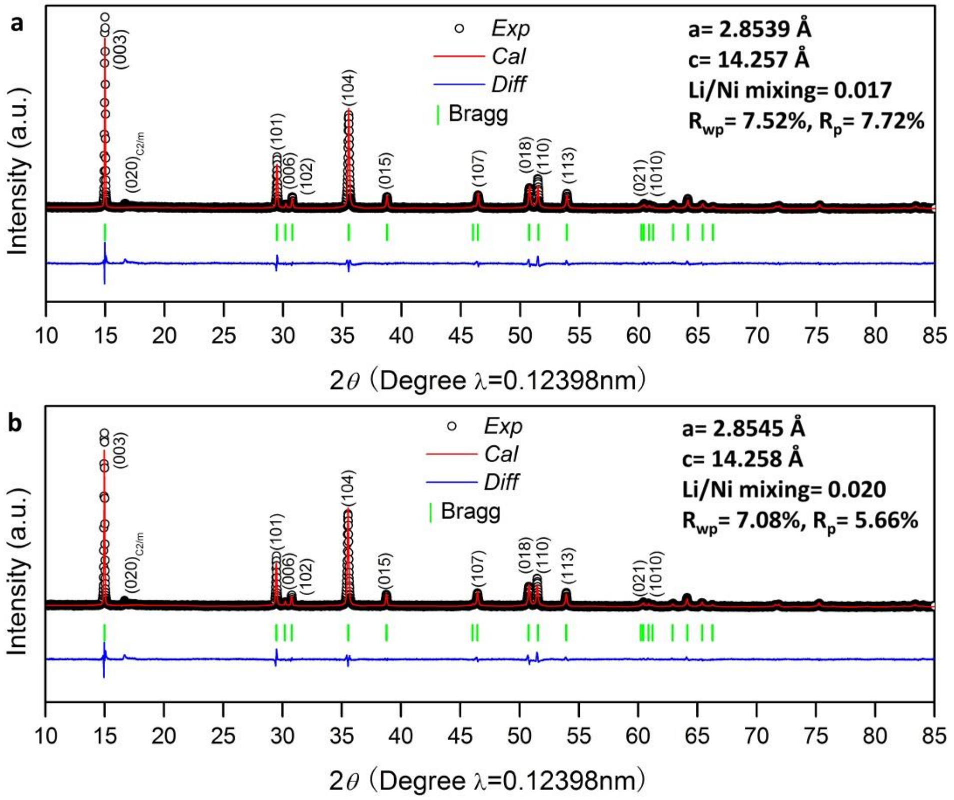

Supplementary Figure 2| Structural characterization of the pristine and GSIR LR-NCM. Synchrotron X-ray diffraction (SXRD) patterns for the pristine (**a**) and GSIR LR-NCM (**b**).

All the peaks in the diffraction patterns presented in Supplementary Fig. 2a and b could be indexed to trigonal space group *R-3m*, whereas the weaker superstructure peaks could be indexed to monoclinic space group *C2/m*. Both lattice parameters and Li/Ni mixing show no big difference between the pristine and GSIR LR-NCM sample. This finding implies that the surface modification does not strongly influence the whole structure.

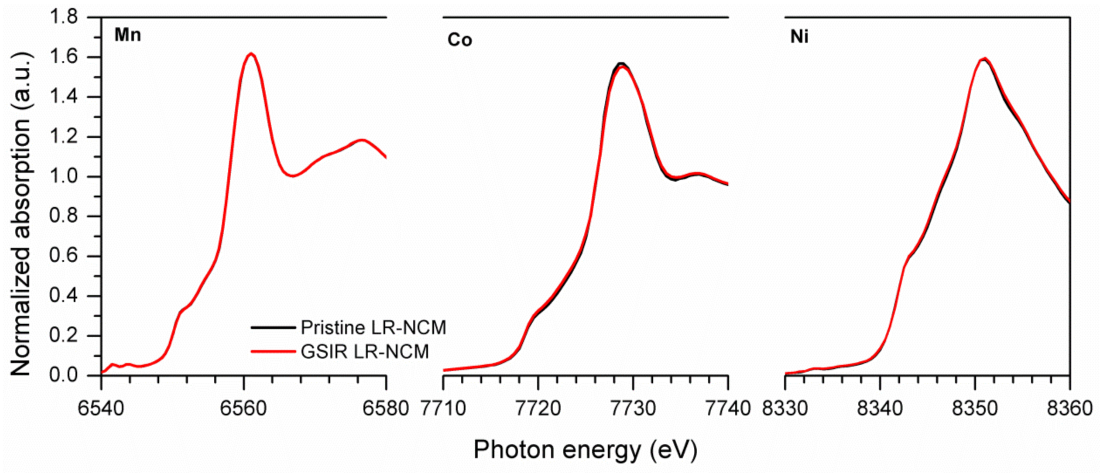

Supplementary Figure 3| Structural characterization of the pristine and GSIR LR-NCM. Normalized K-edge X-ray absorption near edge structure (XANES) spectra for the pristine and GSIR LR-NCM.

As shown in Supplementary Fig. 3, the K-edge XANES data fully overlap each other with little distinction for the pristine and GSIR LR-NCM sample. The results are consistent with the previously published XANES results of this system[2], showing that the average oxidation state is 2+ for Ni, 3+ for Co, and 4+ for Mn. Therefore, the total average and local structure environments for transition metals have not been significantly altered after the GSIR by this novel modification method.

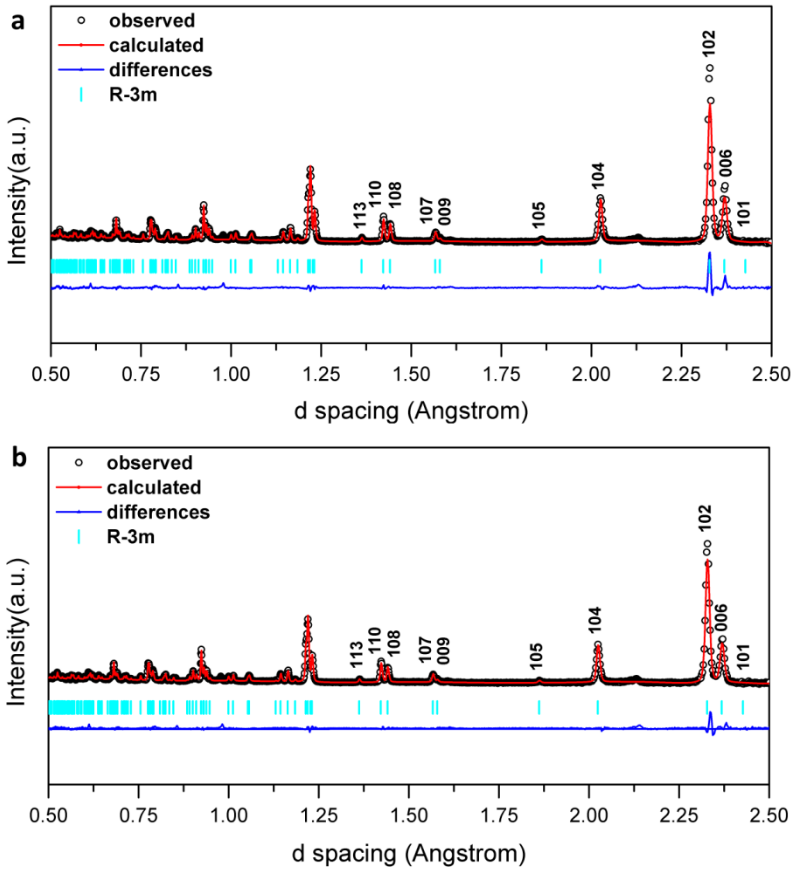

Supplementary Figure 4| Normalized neutron diffraction (ND) patterns for the pristine and GSIR LR-NCM. a. Pristine LR-NCM; **b.** GSIR LR-NCM.

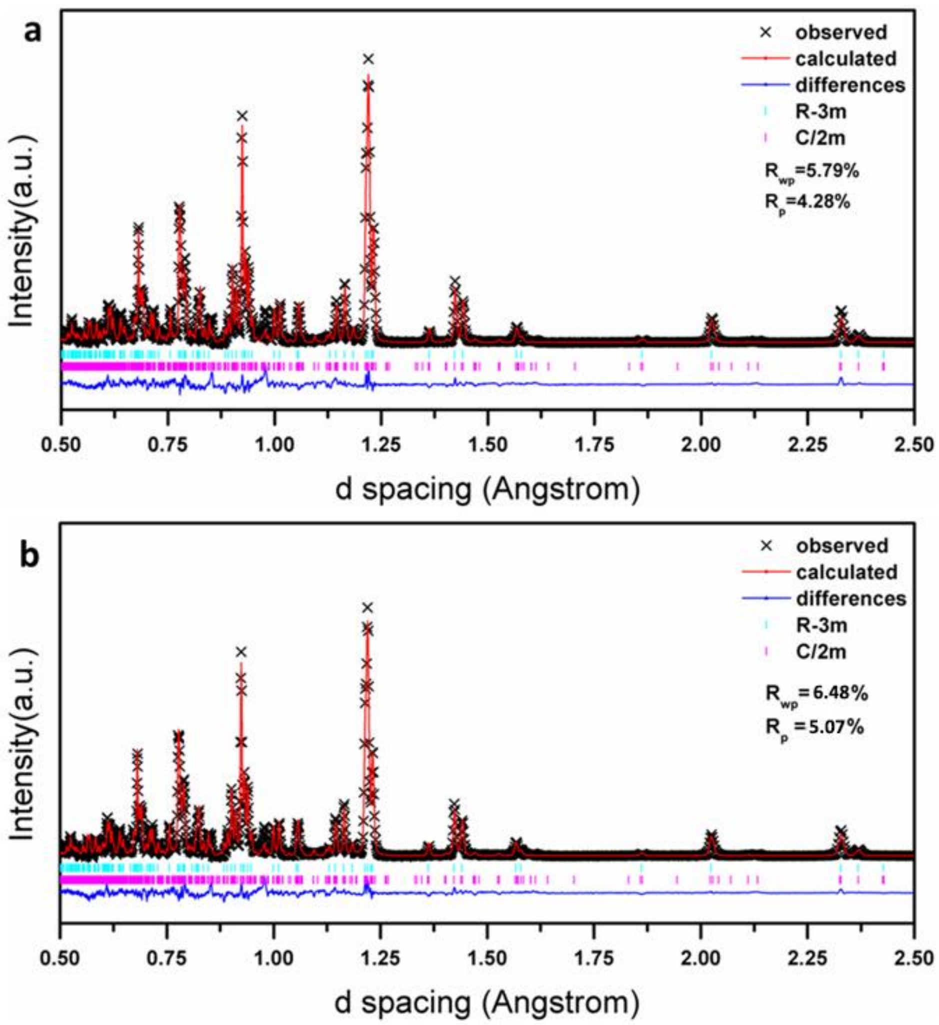

Supplementary Figure 5| Refined powder neutron diffraction for the pristine and GSIR LR-NCM. a. Pristine LR-NCM; **b.** GSIR LR-NCM. *Rietveld* Refinement by considering a composite monoclinic Li_2MnO_3 (C2/m) and rhombohedral $LiMO_2$ (M = Co, Mn, Ni, R-3m) unit cell.

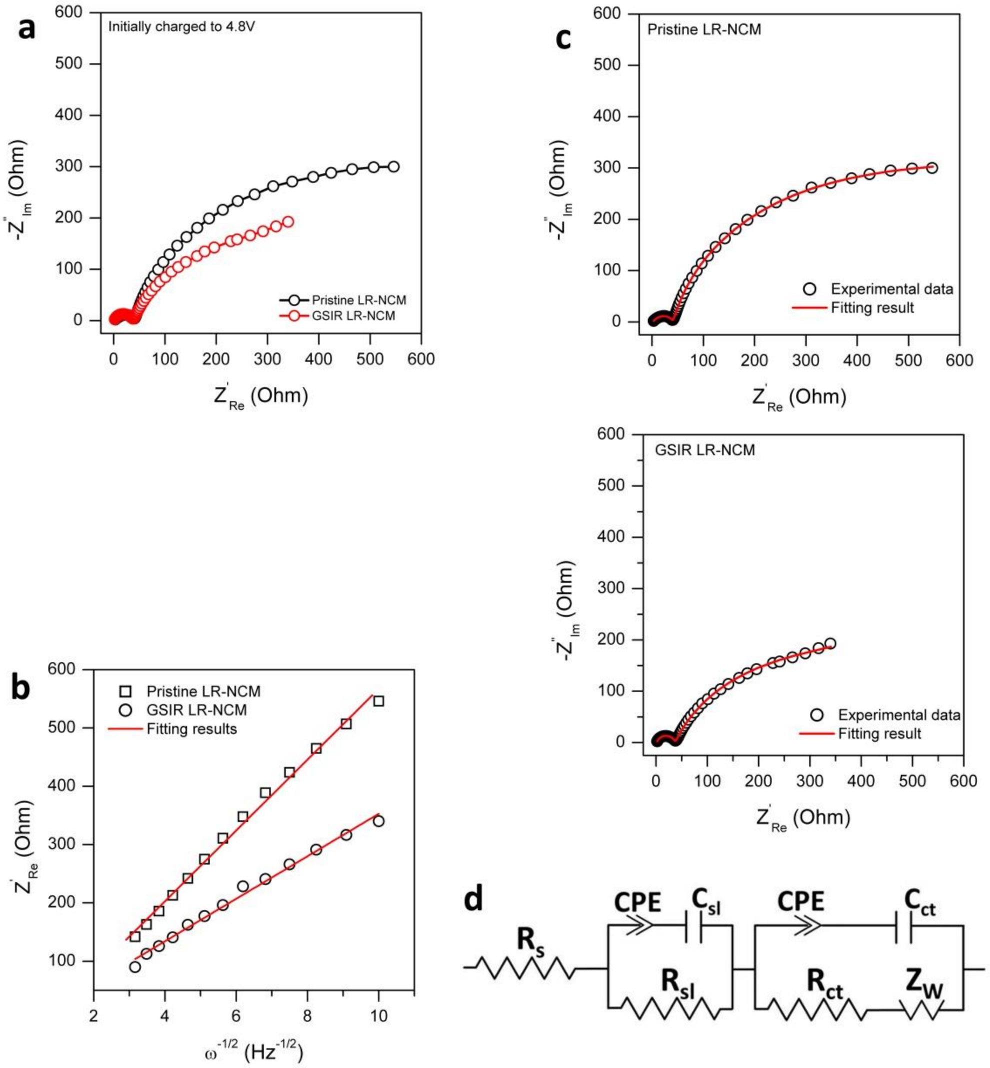

Supplementary Figure 6| Changes of cell impedance for the pristine and GSIR LR-NCM. a. Nyquist plots of the cells with the pristine and GSIR LR-NCM after initially charged to 4.8 V at 0.05 C-rate and rested at 3h; **b.** The profiles of Z'_{Re} *vs.* $\omega^{-1/2}$ from 0.1 Hz to 0.01 Hz after initially charged to 4.8 V; **c.** Typical Nyquist and fitting plots for the pristine and GSIR LR-NCM using the equivalent circuit; **d.** the equivalent circuit.

In the equivalent circuit[3, 4] (Supplementary Fig. 6d), R_s refers to the uncompensated ohmic resistance between the working electrode and the reference electrode; R_{sl} represents the resistance for lithium ion diffusion in the SEI layer; *CPE* and C_{sl} refer to the nonideal capacitance of the surface layer and the surface layer capacitance; R_{ct} and C_{ct} refer to the charge transfer resistance and the charge transfer capacitance; Z_w represents the Warburg impedance describing the lithium ion diffusion in the bulk material. Among these parameters, R_s, R_{ct}, and Z_w can be used to

quantify the ohmic polarization, charge transfer polarization and diffusion polarization. The fitted impedance parameters are listed in Supplementary Table 1. It can be seen that the R_{sl} and R_{ct} for the GSIR LR-NCM is lower than that of the pristine one.

In addition, electrochemical impedance spectroscopy (EIS) is also an important tool for evaluating the diffusion coefficient of a lithium ion within the particles. The diffusion coefficient of the lithium ion is calculated according to the following equation[5, 6]:

$$D_{Li^+} = \frac{R^2T^2}{2A^2n^4F^4C^2\sigma^2} \tag{1}$$

where D_{Li} represents the diffusion coefficient of the lithium ion, *R* is the gas constant, *T* is the absolute temperature, *A* is the surface area of electrode (Based on the BET area), *n* is the number of charges on the Li ion per molecule during oxidization, *F* is the Faraday constant, *C* is the concentration of lithium ion, and σ the Warburg factor, *σ* relates to Z'_{Re} through eqn. (2):

$$\boldsymbol{Z'_{Re} = R_D + R_l + \sigma\omega^{-1/2}} \tag{2}$$

Its value can be obtained from the slope of the lines between Z'_{Re} and $\omega^{-1/2}$ as shown in Supplementary Fig. 6b.

Based on the eqn. (1), the diffusion coefficient of the lithium ion in pristine and GSIR LR-NCM, after initially charged 4.8 V, is only related to the parameter *σ*. Therefore, the diffusion coefficient D_{Li} for GSIR LR-NCM exhibits an increase compared to that of pristine LR-NCM.

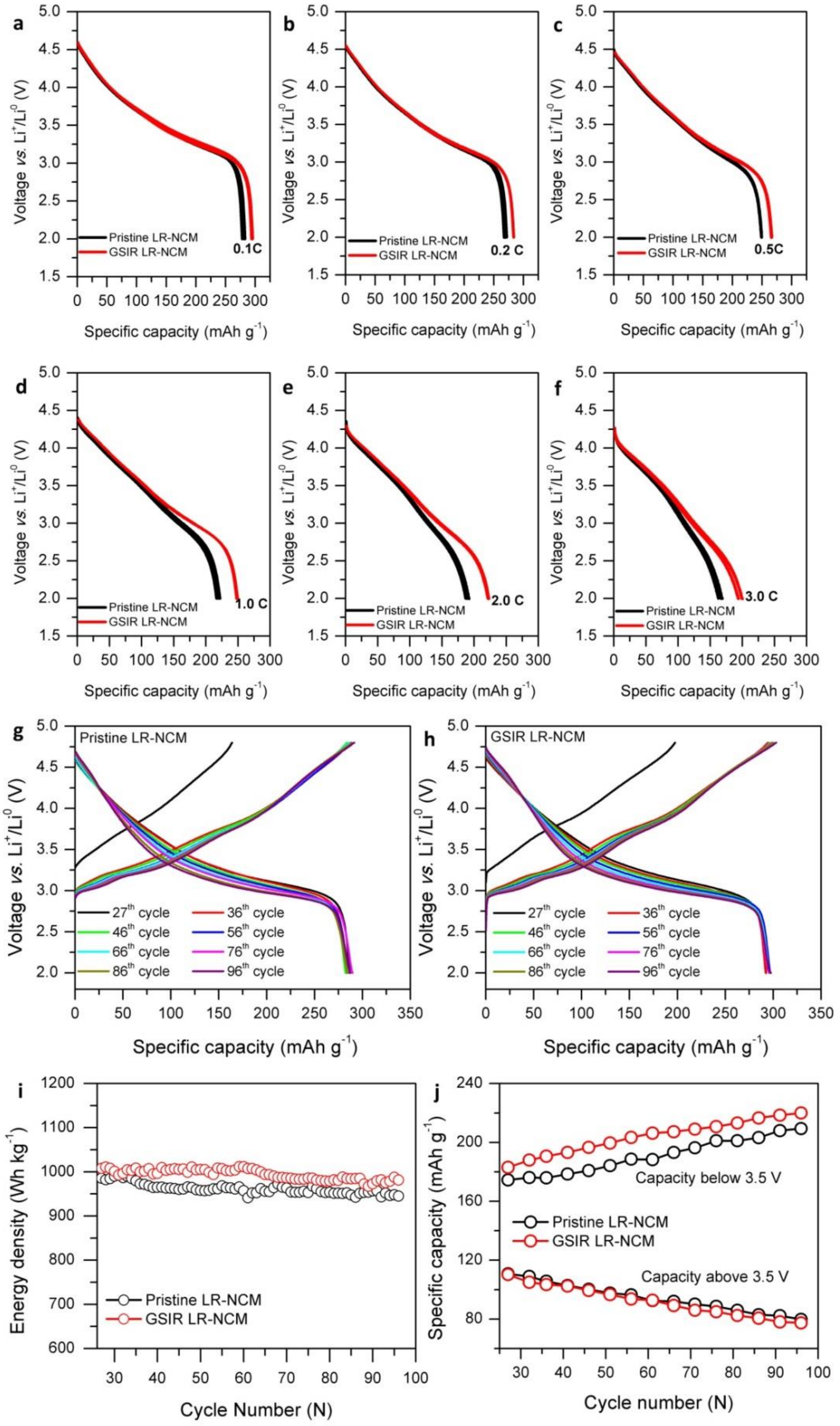

Supplementary Figure 7| Charge-discharge curves of the pristine and GSIR LR-NCM. **a-f.** Comparisons with discharge curves at different rates. The unique change after modification is that the additional rate capacity results from the contribution at low voltage; **g**-**h.** Charge-discharge plots after rate capability. The unique characteristic is

that the voltage decay occurs gradually, while the discharge capacity nearly keeps stable during the subsequent cycles. This voltage profile decay has been considered as the layered-to-spinel transformation[7, 8]. **i.** Discharge energy density as a function of cycle number during the cycling at 0.1 C; **j.** Discharge capacity above 3.5 V and below 3.5 V *vs.* Li^+/Li^0 of the pristine and GSIR LR-NCM during the cycling.

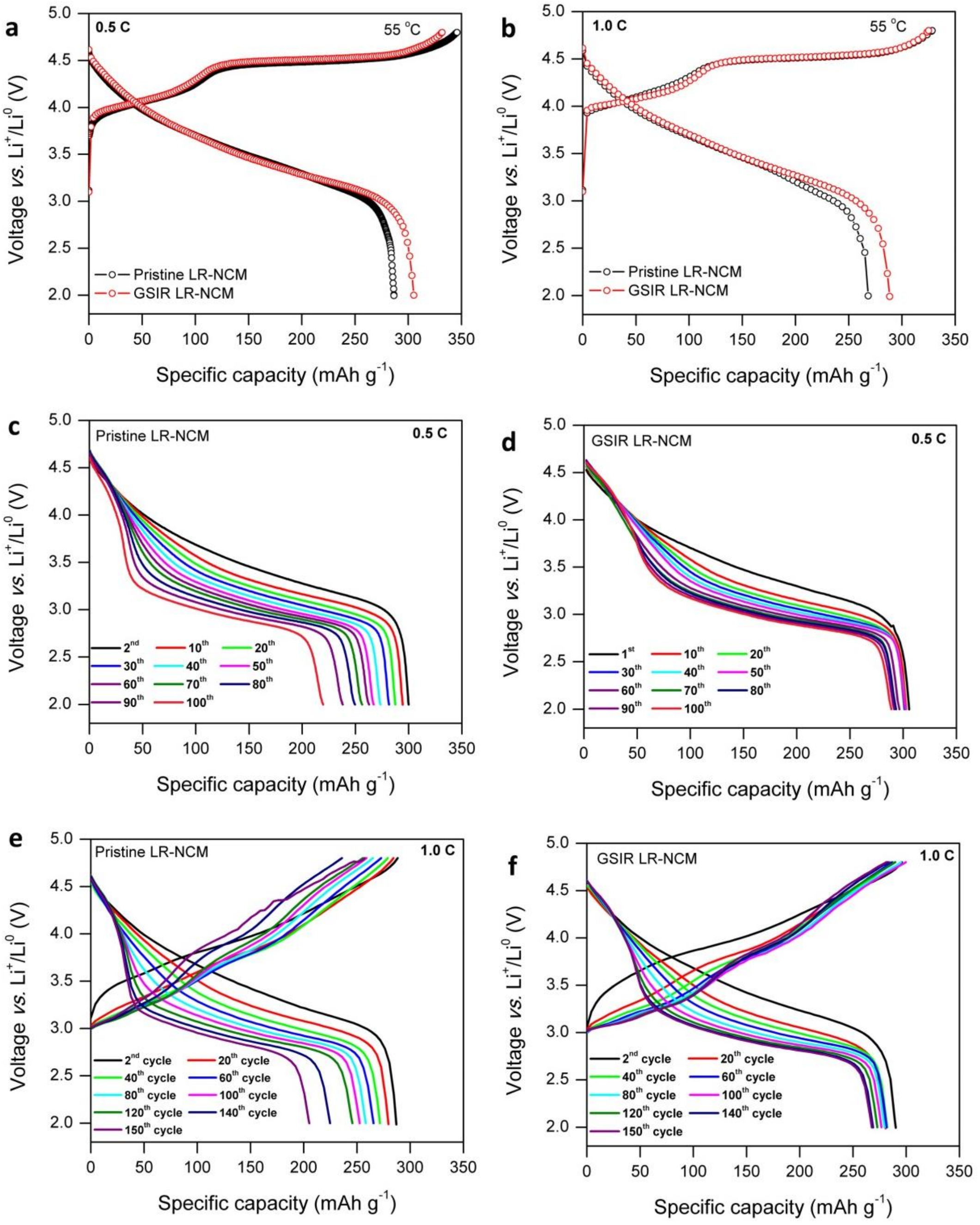

Supplementary Figure 8| Charge-discharge curves of the pristine and GSIR LR-NCM at elevated temperature of 55 °C. a. Initial charge-discharge profiles of the pristine and GSIR LR-NCM at 0.5 C-rate between 2.0 and 4.8 V at 55 °C, where 1.0 C-rate corresponds to the current density of 250 mA g^{-1}; **b.** Initial charge-discharge profiles of the pristine and GSIR LR-NCM at 1.0 C-rate between 2.0 and 4.8V at 55 °C; **c**-**d.** Discharge plots of the pristine and GSIR LR-NCM at 0.5 C-rate cycled between 2.0 and 4.8 V at 55 °C; **e**-**f**. Charge-discharge plots of the pristine and GSIR LR-NCM at 1.0 C-rate cycled between 2.0 and 4.8V at 55 °C.

Supplementary Table 1 Impedance parameters of the pristine and GSIR LR-NCM after initially charged to 4.8 V

	R_e (Ω)	R_{sl} (Ω)	R_{ct} (Ω)	σ
Pristine LR-NCM electrode	1.288	37.72	769.2	60.98
GSIR LR-NCM electrode	1.137	36.44	411.7	36.44

Supplementary Note 1

Analysis of the activity of oxygen

During the operation at high potential, the electrochemical activation of Li_2MnO_3 component is involved with the activity of lattice oxygen in Li-rich layered oxides [9, 10]. In this process, this will lead to generating some oxygen vacancies and oxygen gas simultaneously, especially on the surface.

The formation of oxygen vacancies can be simply represented by:

$$\boldsymbol{O_O} \rightarrow \frac{\boldsymbol{1}}{\boldsymbol{2}}\boldsymbol{O_2} + \boldsymbol{V_{\ddot{O}}} + \boldsymbol{2e'} \qquad (1)$$

Assume that these defects are noninteracting, the law of mass action would be in its simplest form with concentrations instead of activities. In this case, the equilibrium constant K for this reaction is

$$\boldsymbol{K} = [\boldsymbol{V_{\ddot{O}}}][\boldsymbol{e'}]^{\boldsymbol{2}}\boldsymbol{P_{O_2}^{1/2}} \qquad (2)$$

Where the brackets [ ] denote concentrations. Equation (1) shows that the concentrations of oxygen vacancies and electrons are related:

$$[\boldsymbol{e'}] = \boldsymbol{2}[\boldsymbol{V_O''}] \qquad (3)$$

Hence

$$\boldsymbol{K} = \boldsymbol{4}[\boldsymbol{V_{\ddot{O}}}]^3\boldsymbol{P_{O_2}^{1/2}} \qquad (4)$$

Therefore, the oxygen partial pressure of P_{O_2} is given by:

$$\boldsymbol{P_{O_2}} = \frac{\boldsymbol{K}}{\boldsymbol{16}[\boldsymbol{V_{\ddot{O}}}]^{\boldsymbol{6}}} \qquad (5)$$

The oxygen partial pressure is found to be sensitive to the concentration of oxygen vacancies in the lattice, especially on the particle surface.

Based on the above results, the oxygen partial pressure will be less for the sample with surface oxygen vacancies than that of the sample without any oxygen vacancies. Accordingly, this means that the presence of oxygen vacancies on the surface will suppress the generation of oxygen gas.

Supplementary References

1. Yu, H. J. *et al.* Direct atomic-resolution observation of two phases in the $Li_{1.2}Mn_{0.567}Ni_{0.166}Co_{0.067}O_2$ cathode material for Li-ion Batteries. *Angew. Chem. Int. Ed.* **52**, 5969-5973 (2013).
2. Yu, X. Q. *et al.* Understanding the rate capability of high-energy-density Li-rich layered $Li_{1.2}Ni_{0.13}Mn_{0.54}Co_{0.13}O_2$ cathode materials. *Adv. Energy Mater.* **4**, 1300950 (2014).
3. Zheng, J. M. *et al.* The effects of AlF_3 coating on the performance of $Li[Li_{0.2}Ni_{0.13}Co_{0.13}Mn_{0.54}]O_2$ positive electrode material for lithium-ion battery. *J. Electrochem. Soc.* **155**, A775-A782 (2008).
4. Guo, S. H. *et al.* Surface coating of lithium-manganese-rich layered oxides with Delaminated MnO_2 nanosheets as cathode materials for Li-ion batteries. *J. Mater. Chem. A*, **2**, 4422-4428 (2014).
5. Luo, D. *et al.* A new spinel-layered Li-rich microsphere as a high-rate cathode material for Li-ion batteries. *Adv. Energy Mater.* **4**, 1400062 (2014).
6. Li, B. H. *et al.* Facile synthesis of $Li_4Ti_5O_{12}$/C composite with super rate performance, *Energy Environ. Sci.,* **5**, 9595-9602 (2012).
7. Zheng, J. *et al.* Mitigating voltage fade in cathode materials by improving the atomic level uniformity of elemental distribution. *Nano Lett,* **14**, 2628-2635 (2014).
8. Gu, M. *et al.* Formation of the spinel phase in the layered composite cathode used in Li-ion batteries. *ACS Nano,* **7**, 760-767 (2013).
9. Lu, Z. & Dahn, J. R. Understanding the anomalous capacity of Li/ $Li[Ni_xLi_{1/3-2x/3}Mn_{2/3-x/3}]O_2$ cells using *in situ* X-ray diffraction and electrochemical studies. *J. Electrochem. Soc.* **149**, A815-A822 (2002).
10. Armstrong, A. R. *et al.* Demonstrating oxygen loss and associated structural reorganization in the lithium battery cathode $Li[Ni_{0.2}Li_{0.2}Mn_{0.6}]O_2$. *J. Am. Chem. Soc.* **128**, 8694-8698 (2006).
